# Implications of non-native species for mutualistic network resistance and resilience

**DOI:** 10.1371/journal.pone.0217498

**Published:** 2019-06-11

**Authors:** Clare E. Aslan

**Affiliations:** 1 Landscape Conservation Initiative, Northern Arizona University, Flagstaff, Arizona, United States of America; 2 Conservation Science Partners, Flagstaff, Arizona, United States of America; University of Potsdam, GERMANY

## Abstract

Resilience theory aims to understand and predict ecosystem state changes resulting from disturbances. Non-native species are ubiquitous in ecological communities and integrated into many described ecological interaction networks, including mutualisms. By altering the fitness landscape and rewiring species interactions, such network invasion may carry important implications for ecosystem resistance and resilience under continued environmental change. Here, I hypothesize that the tendency of established non-native species to be generalists may make them more likely than natives to occupy central network roles and may link them to the resistance and resilience of the overall network. I use a quantitative research synthesis of 58 empirical pollination and seed dispersal networks, along with extinction simulations, to examine the roles of known non-natives in networks. I show that non-native species in networks enhance network redundancy and may thereby bolster the ecological resistance or functional persistence of ecosystems in the face of disturbance. At the same time, non-natives are unlikely to partner with specialist natives, thus failing to support the resilience of native species assemblages. Non-natives significantly exceed natives in network centrality, normalized degree, and Pollination Service Index. Networks containing non-natives exhibit lower connectance, more links on average, and higher generality and vulnerability than networks lacking non-natives. As environmental change progresses, specialists are particularly likely to be impacted, reducing species diversity in many communities and network types. This work implies that functional diversity may be retained but taxonomic diversity decline as non-native species become established in networks worldwide.

## Introduction

Global environmental change alters both the composition and dynamics of ecological communities, with the potential to disrupt or erode critical ecological functions [1,2]. According to resilience theory, ecosystems undergoing significant changes in species composition may enter alternative stable states, wherein they exhibit fundamental shifts in character with potential associated losses in ecosystem functions and services [3]. Resistant systems can absorb substantial change without transitioning in state, and resilient systems can return to their original state after disruption [4].

Clear definitions of ecosystem state can be elusive [5], so a state change can also be hard to definitively describe. However, in concept, resistance and resilience can be distinguished by degree. Resistance implies that disturbance has occurred but the system retains functions and remains in its current character. This suggests that such systems retain at least full functional diversity; functional redundancy can increase the likelihood that dominant functions persist through disturbance. Resilient systems exhibit a temporary loss of functions and character, but recover that original character in a reasonable length of time [4]. Although these definitions carry an abstract quality that can be problematic [6], I here thus consider resistance to be bolstered by retention of dominant species establishing the major functions of the system. I consider resilience to be bolstered by retention of species richness, as a maximum diversity of life history traits and interactions can promote disturbance recovery and succession following state change [7].

Biological invasions are a primary driver of environmental change [8,9] and have been shown to impact ecological functions from pollination to nutrient cycling to fire regimes [10–12]. Successful, established non-native species become integrated into ecological communities and thus interaction networks that carry out key functions [13,14]. Understanding the consequences of community invasion for ecosystem resilience and resistance requires an understanding of the roles of non-natives in these networks and their functions [15].

Because they lack coevolutionary history where they are introduced, non-native species establishing novel interaction partnerships should be most likely to display generalist behaviors, morphology, and physiology. These interactions should be most likely to involve native partners with similarly generalist traits [13,16,17]. Because of these limitations, novel interactions are unlikely to be evenly distributed across invaded communities [18]. The resistance and resilience of the resulting altered ecosystems, or whether they are able to retain their species assemblages and ecological functions in the face of continued environmental change, will be key to the persistence of native biodiversity going forward [19,1,20].

Species interaction networks have garnered increased attention in recent years as quantitative depictions of interacting ecological communities comprising multiple taxonomic levels [21,22]. By assembling observations of interactions into networks, it becomes possible to analyze indirect interactions among individual taxa, the roles of various species and functional groups in the community (e.g., [23]), and the influence of certain combinations of species on community stability over time [24–30]. Non-native species have entered species interaction networks around the world [31–35]. Non-natives may disrupt existing interactions and threaten biodiversity [13]. At the same time, because successful non-natives demonstrate wide environmental tolerances [36], non-natives in interaction networks have the potential to allow important interaction functions to persist in spite of ongoing environmental change.

Previous studies have used network analysis to investigate the role of non-native species in mutualistic communities, shedding light on patterns that were pivotal to the development of the hypotheses tested here. Both Stouffer et al. [14] and Albrecht et al. [15] selected and compared pollination networks with and without non-native species, analyzing the structure of invaded and uninvaded networks to identify consistent patterns associated with the presence of non-native species. In an analysis of 25 invaded and 34 uninvaded networks, Stouffer et al. [14] determined that pollinators in the networks were disproportionately likely to interact with non-native plants. The authors proposed that non-native plants may thus be particularly beneficial for native pollinators [14] Albrecht et al. [15] analyzed 20 pairs of networks, each consisting of one invaded and one uninvaded network, and found that non-native plants partner with larger numbers of pollinators than do native plants, increasing module size across invaded networks. They also determined that secondary species extinction probability was reduced for invaded networks compared with uninvaded networks [15]. Furthermore, Albrecht et al. [15] proposed that non-native species boost the robustness of networks by acting as generalists and linking species across the network. Both of these studies examined networks post-invasion, assuming that such networks had been rewired following the invasion and examining network structures following that rewiring and in contrast with uninvaded networks.

A more species-focused approach was taken by Emer et al. [35], who examined a set of species occurring in described networks in both their exotic and native ranges. The analysis found that species largely retained the same roles before and after introduction; that is, highly generalist species in their native range are also highly generalist in their invaded ranges [35]. Particularly if the non-natives are generalists and liable to interact in mutualisms with native species, efforts to remove such non-natives might remove critically important species from networks [37] and impact linkage across the networks.

**Fig 1:**
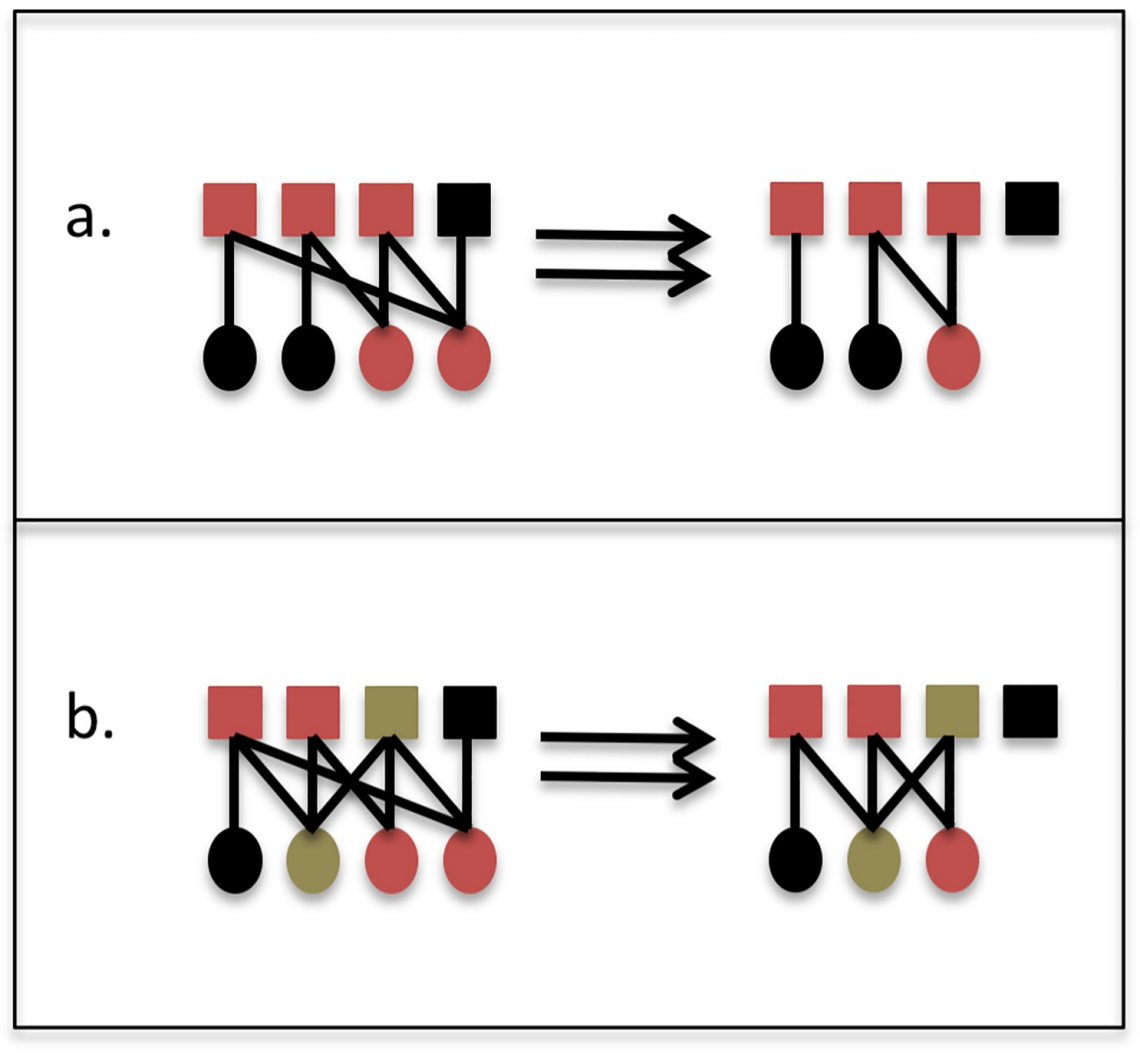
Conceptualization of the relationship between non-native species and network resistance and resilience. a. When no non-natives are present, the hypothetical network contains some generalist native species (shown in red) with multiple partners, and two specialist species (shown in black) with a single partner each. When an extinction occurs, one species and one pair of species become detached from the network. b. When non-native species (delineated in gold) are integrated into the network, they are likely to be more generalist than the average native species, each interacting with multiple partners. When an extinction occurs, one species becomes detached from the network because it was a specialist and the generalist non-natives did not partner with it; this results in an irreversibly altered species assemblage. However, the rest of the network is more robust than the natives-only network, and further network deterioration is unlikely. The functional contributions of the network (e.g., pollination) will persist in their current state, since the broader network structure remains, indicating resistance. At the same time, there has been a loss of species richness, implying reduced resilience; the network contains fewer unique species and life history traits, and this reduced diversity could hinder succession and disturbance recovery in the future.

The hypotheses explored here emerged from the work of these previous studies, applying their insights to investigate the implications of non-native species invasion for mutualistic network resistance and resilience. As non-native species invasions escalate, it has become apparent that generalist species, which interact with a diversity of partners, are particularly likely to successfully enter communities [13,38,16,39]. Non-native species also lack coevolved enemies, so may exhibit high population densities (e.g., [40]). Thus, non-natives may interact with large numbers of partners due simply to their generality and abundance [41]. I therefore hypothesized that, as generalists able to form linkages with many partners [15,14,35], non-native species have become disproportionately central in current network structure and function. I also hypothesized that the presence of non-native species is likely to boost the current resistance of mutualistic networks to future disturbances. Those non-natives are likely to partner with a wide diversity of native species and exhibit high environmental tolerance themselves, thus offering functional insurance under continued environmental change (Fig 1).

## Materials and methods

I used standard network analysis and quantitative research synthesis, coupled with extinction simulations, to compare the network roles of native and non-native species in published, empirical networks. I restricted my analyses to pollination and seed dispersal networks because they have been described sufficiently to permit well-replicated, species-level analyses. I used only mutualistic networks that were published in their entirety or provided directly by authors and contained at least one known non-native species.

Network analysis can be used to produce dozens of metrics describing the structure of networks and the roles of participant species. In my analyses, I included those network metrics relevant to non-native species centrality within the network (Table 1) and thus to theoretical network resistance and resilience. This included four metrics at the species level: normalized degree, betweenness, closeness, and the Pollination Service Index. Because successful establishment of non-native species is most likely for generalists, I expected that non-natives in mutualistic networks would exhibit greater **normalized degree** (number of partners of a given species, relative to the number of possible partners; [41]) than native taxa. I also expected that non-natives would exhibit higher centrality in networks than natives, where centrality may be indicated by **betweenness** (the number of links connecting a species to others in the network; [41]) or **closeness** (the average length of paths connecting the species to each species in the network; [41]). Finally, I expected that, due to their large number of partners, non-native animals in plant-animal mutualistic networks would exhibit on average higher **Pollination Service Index** (the importance of a given animal as a partner for all plant species in the network; [41]) than native animals.

**Table 1:** Network analysis metrics relevant to the centrality and importance of non-native species within networks and included in the analyses performed here.

Metric	Definition	Interpretation
Normalized degree	Species-specific metric; quantifies the total number of partners of a given species, relative to the number of possible partners.	Standardized by network size; non-native species with higher normalized degree than natives are more linked within the network.
Betweenness	Species-specific; the raw number of links connecting a species to others in the network.	Raw value indicating the number of partners of each species; non-native species are more generalist if they exhibit higher betweenness than native species within the same network.
Closeness	Species-specific: the average length of paths connecting the species to each species in the network.	Non-native species are more central than native species if they exhibit higher closeness, which indicates fewer degrees of separation between them and all other species in the network.
Pollination Service Index	Animal-specific; the importance of a given animal as a partner for all plant species in the network.	Non-native animals are more important in the network than natives if their average PSI is higher, indicating that the full suite of plants in the network interacts more with the non-natives than natives.
Connectance	Network-wide; the realized proportion of all possible links between animals and plants.	Higher connectance indicates that a greater proportion of potential links between partners are observed; however, this metric must be used with caution since connectance is affected by network size.
Mean number of links	Network-wide; the average number of links per species across the network.	This raw value provides the mean number of partners per species across the network. If non-native species are more connected than native species, mean links should be greater for invaded networks.
Generality	Network-wide; the mean number of plant species interacting with each animal species.	This raw value provides the mean number of plants interacting with each animal. If non-native animals are more connected than natives, generality should be higher in invaded networks.
Vulnerability	Network-wide; the mean number of animal species interacting with each plant species.	This raw value provides the mean number of animals interacting with each plant. If non-native plants are more connected than natives, vulnerability should be higher in invaded networks.

Four network-level metrics (connectance, mean number of links, generality, and vulnerability) are also relevant to the centrality of non-native species (Table 1) and potential network resistance and resilience. Based on the expectation that non-natives will partner with more species on average than natives, I expected that plant-animal networks containing non-natives would exhibit higher **connectance**, defined in network analysis as the realized proportion of all possible links between animals and plants; [41]), compared to networks from which generalist non-natives and their links had been removed. Enhanced connectance would suggest a more resistant network, with most species interacting with multiple partners to obtain important functions. In essence, a well-connected network should offer insurance against complete detachment of species from the network following disturbance, local extinctions, or population declines. I also expected that networks containing non-native species would exhibit higher **mean numbers of links** per species, higher **generality** (mean number of plant species interacting with each animal species; [41]) and higher **vulnerability** (mean number of animal species interacting with each plant species; [41]) than simulated networks from which non-natives had been removed.

### Network identification

In July 2014, to identify published networks from literature in ISI Web of Science, I employed the search terms *mutualism* and *network* with each of the following: *exotic*, *alien*, *introduced*, *invasive*, and *non-native*. I examined all resulting papers, along with any online or supplementary material, to determine if they contained described networks. I then examined networks to determine if they were provided with sufficient taxonomic resolution, for both higher- and lower-order species, to distinguish native vs. non-native species. All potentially useful networks were either pollination or seed dispersal networks. I assembled these networks into a database for nativity coding. I additionally added all those pollination and seed dispersal networks included in the NCEAS Interaction Web DataBase (https://www.nceas.ucsb.edu/interactionweb/) that included sufficient resolution to determine species nativity.

I examined each network in turn for species origin (native vs. non-native). I used internet search engines and library resources to search for each taxon. When occurrence records indicated that the taxon originates from or is found largely in the region of the network, that taxon was considered likely native. If the taxon was resolved only to genus, family, or order level, and natives of that taxon are known to occur in the region of the network, I considered the taxon likely native; such poor resolution applied to fewer than 10% of species in any given network. In cases where no information could be found after extensive searching, I attempted to contact authors of the networks for further information. (Most authors were unable to assist me on this point). In the very few (<1%) of taxa for which it was still impossible to obtain any information on nativity, I considered the species “probably native,” on the premise that the taxon is clearly rare enough and limited in geographic range enough that it has not attracted attention of taxonomists or biogeographers. Thus, any species determined to be non-native for this network analysis was known to be such. I reasoned that it was more conservative to incorrectly identify a taxon as native than as non-native, since the analyses were statistically comparing non-natives as a group to natives as a group. Across all examined networks, there was a much larger sample size of native than non-native taxa, and thus I could reasonably predict a larger spread of metric values among natives vs. non-natives; incorrect inclusion of a non-native species in the list of natives would run the risk of only extending this spread even more and reducing the likelihood that non-natives as a group emerged as significantly different from native species, thus bolstering Type II but not Type I statistical error.

In all, my search methods netted 119 networks, of which 39 were presented in literature without sufficient taxonomic resolution to determine species origin. A further 22 networks contained enough resolution to enable thorough research of each taxon but contained no verifiably non-native taxa. The final dataset used for my analyses therefore consisted of 58 networks containing known non-native taxa (S1 Table and S1 Fig). This set included 40 pollination and 18 seed dispersal networks from around the globe. (One of these seed dispersal networks, described by [42], was a small island network heavily dominated by non-native species. For some of the analyses detailed below, I removed non-native species to examine network structure in their absence; this reduced the [42] network to only a single seed disperser and the plants with which it interacted, making the network too small for the null model comparisons and extinction simulations described below. As a result, I excluded [42] from those calculations, as indicated by a reduction of degrees of freedom of 1).

### Network analysis and extinction simulations

Once all networks were coded for nativity, I performed network analyses using the R v. 3.2.2 program *bipartite* [43]. I used the functions >networklevel(NETWORK) and >specieslevel(NETWORK) to obtain the full set of network and species metrics for each analyzed network. Because most of the networks were available as only interaction presence/absence data, I was not able to include strength of interactions in these analyses [44]. I extracted the metrics I hypothesized to be associated with species origin (normalized degree, betweenness, closeness, and Pollination Service Index at the species level) to a separate dataframe in which I included each taxon’s origin (native vs. non-native). I then removed all non-native species from the networks and analyzed the resulting natives-only networks to determine whether loss of non-native species resulted in altered network structures. This step constructed artificial networks, but permitted me to evaluate the proportional contribution to each metric of the network species, both native and non-native, by determining whether network structure is significantly altered by non-native species removal. Simulations also provide a glimpse into near-term effects of non-native species eradications, prior to any eventual rewiring. I calculated all network-level analyses for these reduced networks (connectance, mean number of links, generality, and vulnerability) and extracted focal metrics from full and reduced networks to a separate dataframe to compare metrics for those two groups.

However, in nature, sudden removal of non-natives is likely to be followed by some sort of rewiring, increase in native densities, or appearance of new interactors. To control for the effect of the changed numbers of species resulting from removal of non-natives (which has been shown to influence connectance in particular, with larger networks demonstrating reduced connectance; [25,45,37]), I additionally analyzed control networks developed by removal of native taxa equal in number to non-natives in each network. I developed these controls using a random number generator to select native species in each trophic level to remove from the full, unaltered network until I had removed the same number of species per level, with their linkages, as had been removed to generate native-only networks. Note that in most cases the number of species removed per network following this procedure was small (<3 species). I calculated the same network-level analyses for these removal control networks as for the original and reduced networks described above.

As an additional control that did not retain the original linkages of the network, I used the *nullmodel* function in *bipartite*, in conjunction with the *extinction* function, to generate simple null models following extinction of an equal number of species from each network as there were non-natives (to hold the total number of lower-order and higher-order species the same), drawing species randomly for extinction and generating 1000 repetitions of each null model. I used the mean network-level connectance, links per species, generality, and vulnerability from these null models to compare networks with exotics removed to the null models generated from the same networks, in order to determine whether the effect of removing non-native species differed from models that ignored species identity. I also simulated secondary extinctions, removing non-native species and comparing the number of resulting secondary extinctions to those emerging when an equivalent number of native species selected at random were removed. In all simulations, three species on average were removed from each trophic level (out of an average total network size of 79.7 species), thus leaving much of the original network intact.

### Species-level and network-level analyses

I employed linear mixed effects models to determine whether significant differences existed in the species-level metrics between native and non-native taxa across the full set of analyzed networks. I also examined the effect of network type (pollination vs. seed dispersal) as an initial blocking factor, but found no significant effect of type so pooled networks across type for further analyses. The final tested models included the study containing the network as a random blocking effect, taxonomic level (i.e., whether the taxon was an animal or plant) as a fixed effect, and whether the taxon was a known exotic as a fixed effect. Response variables for these models, in turn, included normalized degree, betweenness, closeness, and PSI. For network-level analyses, I began by performing paired two-tailed Student’s t-tests to compare the full set of full networks with each network after exotics had been removed. Response variables, in turn, included connectance, mean number of links, generality, and vulnerability. I repeated this procedure to compare full networks with networks after random removals and to compare networks following exotic removal with null model networks.

These simulations in effect treated all species as equivalent, although in nature some species are known to exert particularly strong influence on network architecture (e.g., [34]). Within the subset of analyzed pollination networks in which it appeared (n = 14), I therefore separately explored the effect on network structure of removal of the supergeneralist European honeybee (*Apis mellifera*), a species non-native in most geographic regions that is known to interact with many native species worldwide [46].

To simulate extinctions of non-native species, I manually removed all non-natives from networks and determined the number of remaining species that now lacked partners, considering those “secondary extinctions.” (Note: this is a common method of quantifying the risk that a species loses all partners, but translation of this to secondary extinction in the real world assumes often unrealistic dependence on the interaction such that its lack results in eventual extinction of the remaining partner (e.g., [47,48,49]). I used the second.extinct function in *bipartite* to graph secondary extinctions following random losses from each network, setting the number of replicate random secondary extinction simulations at 1000. I used the resulting function to determine the number of secondary extinctions to be expected following an equivalent number of random losses as the number of non-native species in each network. I then used a paired two-tailed Student’s t-test to compare the number of secondary extinctions following exotic vs. random losses from each network. For all statistical tests, I tested assumptions of the underlying model by plotting residuals to ensure error independence and normality.

## Results

**Fig 2:**
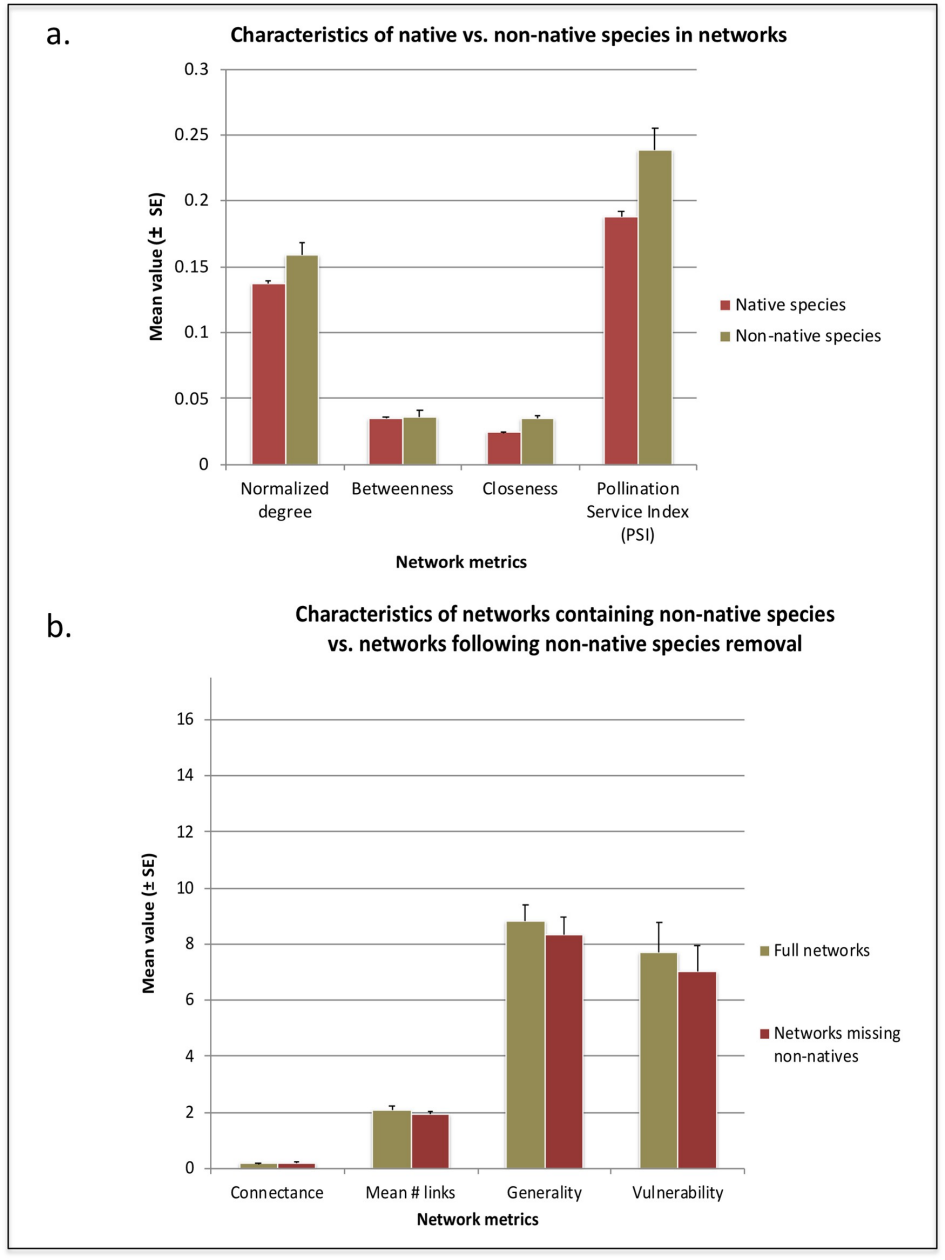
Mean network metrics for analyses of native and non-native species in pollination and seed dispersal networks. a. Species-level analysis results. b. Network-level analysis results. Results were obtained via a research synthesis and extinction simulations for 58 described mutualistic networks.

Species level analyses found that natives and non-natives differed significantly in measured species-level metrics, when network was employed as a blocking factor. Non-native species exceeded native species in normalized degree (t = 2.0053; p = 0.0450) (Fig 2a), consistent with the hypothesis that non-natives would interact with a higher proportion of possible partners than would natives. Non-natives also exhibited significantly higher betweenness (t = 3.6408; p = 0.0003) and closeness (t = 3.0762; p = 0.0021) (Fig 2a). Finally, PSI was significantly higher for non-native animals (0.24 ± 0.016 SE) than for native animals (0.19 ± 0.0048 SE) (t = 4.1368; p < 0.0001) (Fig 2a).

Network-level analyses were consistent with species-level results and permitted comparison with reduced and null networks. Networks including non-natives exhibited significantly lower connectance (0.19 ± 0.016 SE) than networks after non-native species removal (0.21 ± 0.018 SE) (t = 4.3639; p < 0.0001; df = 57) (Fig 2b; Table 2). Mean links per species was significantly higher for full networks (2.09 ± 0.12 SE) than for networks with non-native species removed (1.94 ± 0.12 SE) (t = 4.3658; p < 0.0001; df = 57) (Fig 2b; Table 2). When an equal number of native species were removed at random, connectance also exceeded that of full networks (0.21 ± 0.018 SE) (t = 4.2981; p < 0.0001; df = 57), but the mean number of links (2.19 ± 0.25 SE) did not differ between full networks and networks after random removals (Table 2). On average, as is to be expected given the species-level results, these network metrics indicate that non-native species thus exhibit a higher number of partners per species than the average native species. However, non-natives fail to significantly increase the total proportion of links realized.

**Table 2:** Network-scale metric comparisons of full empirical networks with each of the following: (1) reduced networks from which non-natives were removed; (2) reduced networks from which taxa equivalent in number to the non-natives were removed at random; (3) simple null model reduced networks.

	Full networks	Native-only network			Random removal control			Null control		
Metric	Mean (+/- SE)	Mean (+/- SE)	t*	*P*	Mean (+/- SE)	t*	*P*	Mean (+/- SE)	t*	*P*
Connectance	**0.19 ± 0.016**	0.21 ± 0.018	4.3639	< 0.0001	0.21 ± 0.018	4.2981	< 0.0001	0.25 ± 0.027	3.037	0.0036
Mean links	**2.09 ± 0.12**	1.94 ± 0.12	4.3658	< 0.0001	2.19 ± 0.25	0.4203	0.6759	2.11 ± 0.14	0.0151	0.988
Generality	**8.80 ± 0.62**	8.33 ± 0.63	2.6139	0.0114	7.91 ± 0.63	3.6814	0.0005	7.35 ± 0.54	3.551	0.0008
Vulnerability	**7.72 ± 1.03**	7.00 ± 0.96	3.177	0.0024	7.10 ± 0.96	3.6429	0.0006	7.35 ± 0.54	0.3856	0.7013

*Paired, two-tailed t-statistics refer to comparison of each modified network with the original, empirical, full network containing both native and non-native species.

When non-native species were removed from networks, generality was significantly reduced (8.33 ± 0.63 SE) compared with full networks (8.80 ± 0.62 SE) (t = 2.6139; p = 0.0114; df = 57) (Fig 2b), as was vulnerability (7.00 ± 0.96 SE) compared with full networks (7.72 ± 1.03 SE) (t = 3.177; p = 0.0024; df = 57) (Fig 2b; Table 2). Both of these results, consistent with species-level results, again indicate that the average non-native species exhibits a higher number of partners in these networks than the average native species.

Null model networks were equal in size to networks following non-native species removals, an important consideration since connectance is affected by the total number of species in each network. Null models exhibited significantly higher connectance (t = 2.143; p = 0.0366; df = 56) and lower generality (t = 2.075, p = 0.0427, df = 56) than networks following non-native species removals and exhibited similar results when compared with full networks (for connectance, t = 3.037, p = 0.0036, df = 56; for generality, t = 3.551, p = 0.0008, df = 56) (Table 2).

When I compared metrics of networks that had *A*. *mellifera* as a non-native species (n = 14) with the same networks after *A*. *mellifera* removal, the mean number of links per taxon differed significantly (t = 3.1953; p = 0.0065), with full networks exhibiting more links (mean 1.84 ± 0.18 SE) than networks with *A*. *mellifera* removed (1.76 ± 0.17 SE). For all other metrics, there was no significant difference between networks containing *A*. *mellifera* and networks from which *A*. *mellifera* had been removed.

Across analyzed studies, there were fewer secondary extinctions following losses of non-native species (0.042 ± 0.010 SE) than following simulated random losses (0.11 ± 0.018 SE) (t = 3.8554; p = 0.0003; df = 56). This result is consistent with the premise that non-native species are disproportionately more likely to interact with generalist, well-connected native partners than with specialists; losses of non-native species are disproportionately unlikely to remove the sole link of any specialist native partner.

## Discussion

The non-native species in the analyzed networks appear to strengthen redundancy by interacting with species already well-linked within the networks. This is evidenced by significant differences between native and non-native species in normalized degree, betweenness, closeness, and PSI, as well as higher mean links per species, generality, and vulnerability for full networks than native-only or control networks. Results also suggest that non-native species in these networks rarely partner with specialist species that are poorly-linked within the network, since random extinctions of natives more often remove the sole partner of remaining species than do extinctions of non-native species. In other words, loss of non-natives will most often remove redundant links from networks. Empirical networks in this study were less connected and more generalized than null networks, indicating that a small proportion of species in empirical networks are particularly well-linked and accompany a large number or tail of poorly linked species, relative to taxa in simulated null networks.

*Apis mellifera* is a key non-native generalist pollinator in nearly all parts of the globe. As such, it serves as an ideal focal organism for examination of the potential effect of individual non-natives on networks. Interestingly, the results obtained here indicate that *A*. *mellifera* is exceptionally well-linked in networks, but detect no other consistent architectural influence of the species. Although *A*. *mellifera* has been shown to be particularly central in many networks worldwide [46], these results confirm that it was not by itself responsible for the differences detected between natives and non-natives across the networks analyzed here. Its supergeneralist traits, however, are illustrative of the capacity of certain non-native species to partner with extremely high numbers of species and thus to rewire networks [46].

Across the full set of networks analyzed here, the finding that non-natives contribute to redundancy in interactions suggests that they may in theory bolster network functional *resistance*, defined in resilience ecology as the ability of a system to persist in its current state in the face of environmental disturbance [4]. That is, even if the network loses some of its native members due to future disturbance, non-native species help to ensure that well-linked natives retain partners and that the network itself and thus pollination or seed dispersal as functions will endure (Fig 1).

**Fig 3:**
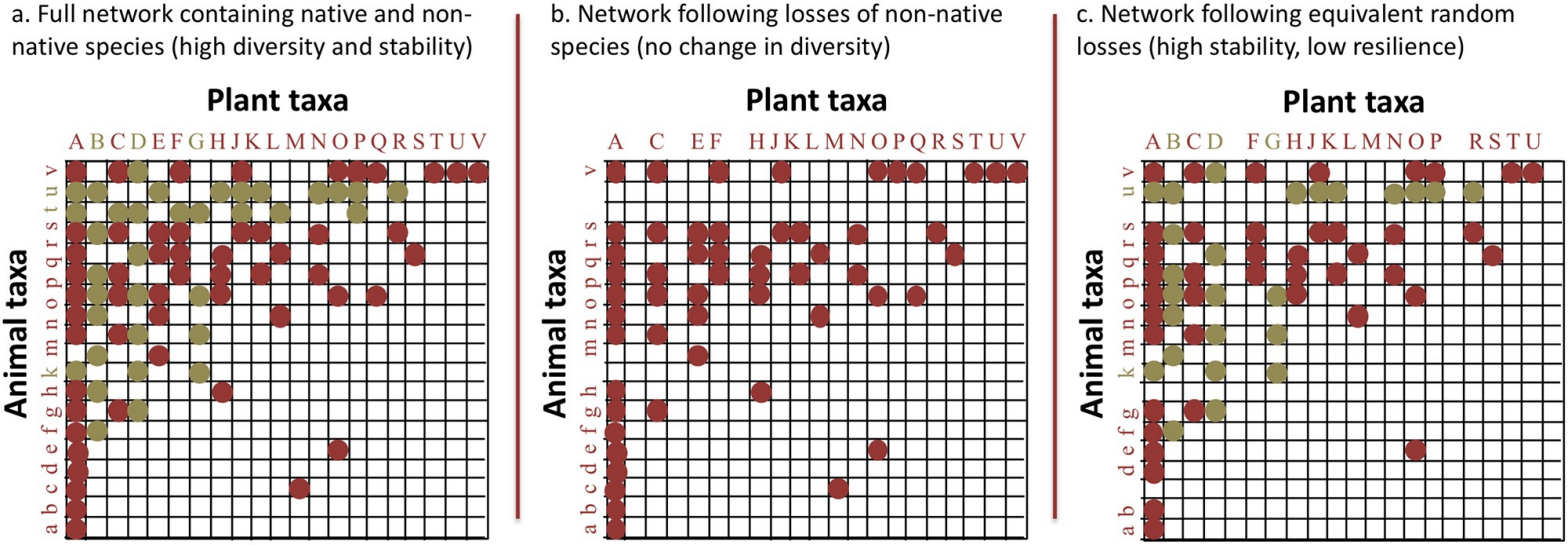
Hypothetical mutualistic network diagrams illustrating the contrasting roles of native and non-native species. a. The full network contains both native (red) and non-native (gold) species. b. When non-native species are removed, the network becomes simplified but no coextinctions have occurred; all natives are still present and retain at least one interaction partner. c. When an equivalent number of species is removed at random from the network, secondary extinctions of specialist native species may occur (as has occurred for plant species M, which now lacks all partners). The network has decreased in native species diversity and is thus less resilient to future disturbance.

At the same time, a theoretical view of resilience would suggest that these non-natives are unlikely to support the resilience of the network’s species assemblage (Fig 3), where resilience is defined as the ability of a system to return to its previous ecological state following perturbation [50]. When environmental change perturbs a community, previous work suggests that specialist species are most vulnerable to extinction [16]. Non-natives provide generalist mutualist functions within a network, but the results of this study as well as previous meta-analyses [14,15,35] are consistent with the premise that non-natives in networks are unlikely to partner with specialist or rare natives or buffer them from disturbance and partner loss. The large majority of native species in the studied networks are poorly-linked and thus considered “specialists,” and these may be vulnerable to secondary extinctions as a result of environmental change.

Previous research has identified mutualistic network characteristics associated with network resistance and resilience [51,27,52]. High complexity and species diversity in mutualistic network simulations bolstered resilience to disturbance [51]. High nestedness and connectance in both theory and meta-analysis supported mutualistic network stability [27,52], although they had the opposite effect in trophic networks [27]. Other studies have found reduced stability of mutualistic networks as nestedness increases [53,54,55]. In theoretical simulations, increased species diversity and nestedness contributed to increased resistance but, as for the empirical studies examined here, reduced resilience due to a high occurrence of rare/specialized species that were prone to extinction following disturbance [51].

By employing empirical networks and examining a specific group of organisms of interest, non-native species, the work described here lends a real-world complement to these previous efforts. In the analyzed networks, drawn from study systems all over the world, non-native and native species in networks are not interchangeable, even within the broad-brush structural modeling realm of network analysis: non-native mutualists are likely to stabilize ecological communities by boosting network functional resistance, but unlikely to interact with specialist members and thus support the full species complement of the native community (Fig 1). In real-world terms, invaded networks may withstand disturbance because their generalists are well-buffered, but are because non-native species are unlikely to partner with and offer services to specialist native species, these networks are probably unlikely to recover their biodiversity following species losses.

Beyond the case study of animal/plant mutualisms examined here, the homogenization of communities as generalists replace specialists is an increasing trend [56,57] that affects all ecological interaction types and can be observed in corollary in social and economic systems, as well (e.g., market globalization; [58]). Understanding whether these changes in a broader set of systems consistently promote resistance and maintain functional diversity at the potential cost of resilience and taxonomic diversity, as discussed here, is a compelling follow-up research direction. Continued efforts to link the insights from resilience theory with network dynamics may assist researchers and managers in efforts to prepare for future trajectories of invaded communities.

## Supporting information

S1 Fig Sources and visualizations of networks included in this research synthesis. In the matrix visualizations, black boxes signify interactions between native and non-native species, gray boxes signify interactions between native species, and white boxes signify missing links.
(DOCX)

S1 Table Sources of empirical networks included in analyses. Note that some sources provided multiple networks, and the number of networks drawn from each source is listed here.
(DOCX)
