# Supplementary material for: Implications of non-native species for mutualistic network resistance and resilience
Source: PLoS One. 2019 Jun 11;14(6):e0217498. doi: 10.1371/journal.pone.0217498 (PMC6559630; doi:10.1371/journal.pone.0217498)
Supplement: S1 Fig — In the matrix visualizations, black boxes signify interactions between native and non-native species, gray boxes signify interactions between native species, and white boxes signify missing links. (DOCX) [file pone.0217498.s001.docx]

**S1 Figure.**

**Figure S1 (*continued*):**

**Figure S1 (*continued*):**

**Figure S1 (*continued*):**

**Figure S1 (*continued*):**

**Figure S1 (*continued*):**

**Figure S1 (*continued*):**

**Figure S1 (*continued*):**

**Figure S1 (*continued*):**

**Figure S1 (*continued*):**

**Figure S1 (*continued*):**

**Figure S1 (*continued*):**


**Figure S1:** Sources and visualizations of networks included in this research synthesis. In the matrix visualizations, black boxes signify interactions between native and non-native species, gray boxes signify interactions between native species, and white boxes signify missing links.
