# Supplementary material for: Implications of non-native species for mutualistic network resistance and resilience
Source: PLoS One. 2019 Jun 11;14(6):e0217498. doi: 10.1371/journal.pone.0217498 (PMC6559630; doi:10.1371/journal.pone.0217498)
Supplement: S1 Table — Note that some sources provided multiple networks, and the number of networks drawn from each source is listed here. (DOCX) [file pone.0217498.s002.docx]

**S1 Table:** Sources of empirical networks included in analyses. Note that some sources provided multiple networks, and the number of networks drawn from each source is listed here.

| Network source | # networks provided |
| --- | --- |
| Aizen MA, Morales CL, Morales JM (2008) Invasive mutualists erode native pollination webs. *PLoS Biology* 6:e31. | 8 |
| Arroyo MTK, Primack RB, Armesto JJ (1982) Community studies in pollination ecology in the high temperate Andes of Central Chile. I. Pollination mechanisms and altitudinal variation. *Amer J Botany* 69:82-97. | 3 |
| Baird JW (1980) The selection and use of fruit by birds in an eastern forest. *Wilson Bull* 92:63-73. | 1 |
| Barrett SCH, Helenurm K (1987) The reproductive biology of boreal forest herbs. 1. Breeding systems and pollination. *Can J Botany* 65:2036-2046. | 1 |
| Bartomeus I, Vilà M, Santamaría L (2008) Contrasting effects of invasive plants in plant–pollinator networks. *Oecologia* 155*:*761-770. | 1 |
| Beehler B (1983) Frugivory and polygamy in birds of paradise. *The* *Auk* 100:1-12. | 1 |
| Bezerra ELS, Machado IC, Mello MAR (2009) Pollination networks of oil-flowers: a tiny world within the smallest of all worlds. *J Animal Ecol* 78:1096-1101. | 1 |
| Carlo TA, Collazo JA, Groom MJ (2003) Avian fruit preferences across a Puerto Rican forested landscape: pattern consistency and implications for seed removal. *Oecologia* 134:119-131. | 4 |
| Carvalheiro LG, Barbosa ERM, Memmott J (2008) Pollinator networks, alien species and the conservation of rare plants: *Trinia glauca* as a case study*. J Appl Ecol* 45:1419-1427. | 1 |
| Crome FHJ (1975) The ecology of fruit pigeons in tropical Northern Queensland. *Aust J Wildlife Res* 2:155-185. | 1 |
| Dicks LV, Corbet SA, Pywell RF (2002) Compartmentalization in plant–insect flower visitor webs. *J Anim Ecol* 71:32–43. | 1 |
| Dupont YL, Hansen DM, Olesen JM (2003) Structure of a plant-flower-visitor network in the high-altitude sub-alpine desert of Tenerife, Canary Islands. *Ecography* 26:301-310. | 1 |
| Elberling H, Olesen JM (1999) The structure of a high latitude plant-flower visitor system: the dominance of flies. *Ecography* 22:314-323. | 1 |
| Galetti M, Pizo MA (1996) Fruit eating birds in a forest fragment in southeastern Brazil. *Ararajuba*, *Revista Brasileira de Ornitologia* 4:71-79. | 1 |
| Hagen M, Kraemer M (2010) Agricultural surroundings support flower–visitor networks in an Afrotropical rain forest*. Biol Conserv* 143:1654-1663. | 3 |
| Hammann A, Curio B (1999) Interactions among frugivores and fleshy fruit trees in a Philippine submontane rainforest. *Conserv Biol* 13:766-773. | 1 |
| Heleno RH, Ramos JA, Memmott J (2013) Integration of exotic seeds in an Azorean seed dispersal network. *Biol Invasions* 15:1143-1154. | 1 |
| Hocking B (1968) Insect-flower associations in the high Arctic with special reference to nectar. *Oikos* 19:359-388. | 1 |
| Jordano P (1985) El ciclo anual de los paseriformes frugívoros en el matorral mediterráneo del sur de España: importancia de su invernada y variaciones interanuales. *Ardeola* 32:69-94. | 1 |
| Jordano P, unpublished data. Hato Ratón, Doñana, SW Spain. | 1 |
| Kaiser-Bunbury CN, Memmott J, Müller CB (2009) Community structure of pollination webs of Mauritian heathland habitats. *Pers Plant Ecol Evol Syst* 11:241–254. | 2 |
| Kevan PG (1970) High arctic insect-flower visitor relations: the inter-relationships of arthropods and flowers at Lake Hazen, Ellesmere Island, Northwest Territories, Canada. Ph.D. thesis, University of Alberta. | 1 |
| McCullen CK (1993) Flower-visiting insects of the Galapagos Islands. *Pan-Pacific Entom* 69:95-106. | 1 |
| Medan D, *et al*. (2002) Plant-pollinator relationships at two altitudes in the Andes of Mendoza, Argentina. *Arctic* *Antarc Alpine Res* 34:233-241. | 2 |
| Motten AF (1986) Pollination ecology of the spring wildflower community of a temperate deciduous forest. *Ecol Mon* 56:21-42. | 1 |
| Muñoz AA, Cavieres LA (2008) The presence of a showy invasive plant disrupts pollinator service and reproductive output in native alpine species only at high densities. *J* *Ecology* 96:459-467. | 1 |
| Olesen JM *et al*. (2010) Missing and forbidden links in mutualistic networks. *Proc Royal Soc London B* 278:725-732. | 1 |
| Olesen JM, Bascompte J, Elberling H, Jordano P (2008) Temporal dynamics in a pollination network. *Ecology* 89:1573-1582. | 1 |
| Olesen JM, Eskildsen LI, Venkatasamy S (2002) Invasion of pollination networks on oceanic islands: importance of invader complexes and endemic super generalists. *Divers Dist* 8:181-192. | 2 |
| Ollerton J, Johnson SD, Cranmer L, Kellie S (2003) The pollination ecology of an assemblage of grassland asclepiads in South Africa. *Ann Botany* 92:807-834. | 1 |
| Percival M (1974) Floral ecology of coastal scrub in southeast Jamaica. Biotropica 6:104-129. | 1 |
| Ramirez N, Brito Y (1992) Pollination biology in a palm swamp community in the Venezuelan central plains. *Bot J Linn Soc* 110:277-302. | 1 |
| Ramirez N (1989) Biología de polinización en una comunidad arbustiva tropical de la alta Guyana Venezolana. Biotropica 21:319-330. | 1 |
| Schemske DW, *et al*. (1978) Flowering ecology of some spring woodland herbs. *Ecology* 59:351-366. | 1 |
| Schleuning M, *et al*. (2011) Specialization and interaction strength in a tropical plant-frugivore network differ among forest strata. *Ecology* 92:26-36. | 1 |
| Silva WR, De Marco P, Hasui E, Gomes VSM (2002) Patterns of fruit-frugivores interactions in two Atlantic Forest bird communities of South-eastern Brazil: implications for conservation. *Seed dispersal and frugivory: ecology, evolution and conservation*, eds Levey DJ, Silva WR, Galetti M (Wallingford: CAB International), pp 423-435. | 1 |
| Small E (1976) Insect pollinators of the Mer Bleue peat bog of Ottawa. *Can Field Nat* 90:22-28. | 1 |
| Snow BK, Snow DW (1972) Feeding niches of hummingbirds in a Trinidad valley. *J Animal Ecol* 41:471-485. | 1 |
| Spotswood EN, Meyer J-Y, Bartolome JW (2012) An invasive tree alters the structure of seed dispersal networks between birds and plants in French Polynesia. *J* *Biogeogr* 39:2007-2020. | 1 |
| Wheelwright NT, Haber WA, Murray KG, Guindon C (1984) Tropical fruit-eating birds and their food plants: a survey of a Costa Rican lower montane forest. Biotropica 16:173-192. | 1 |
| Williams NM (2011) Restoration of nontarget species: bee communities and pollination function in riparian forests. *Rest Ecol* 19:450-459. | 1 |
